# A Laboratory Assessment of Two Local Strains of the* Beauveria bassiana* (Bals.) Vuill. against the* Tetranychus urticae* (Acari: Tetranychidae) and Their Potential as a Mycopesticide

**DOI:** 10.1155/2017/7628175

**Published:** 2017-11-08

**Authors:** Serkan Ortucu, Omer Faruk Algur

**Affiliations:** ^1^Department of Molecular Biology and Genetics, Erzurum Technical University, Erzurum, Turkey; ^2^Department of Biology, Faculty of Science, Atatürk University, Erzurum, Turkey

## Abstract

This study was conducted to assess highly pathogenic* Beauveria bassiana* isolates to be used in biocontrol and to determine their potentials as mycopesticide. For this purpose, two* B. bassiana* isolates, which were locally isolated from* T. urticae*, were chosen. Firstly, three suspensions were investigated at the degree of humidity of 65 ± 5% and 100% RH. Secondly, these strains were selected according to their tendency to mass production, tolerance to UV radiation, and capability of producing spore at the different temperatures. Finally, identification of the selected isolate was performed by using ITS rDNA analysis. Both tested fungal isolates were pathogenic to the* T. urticae*. Mycelial growths of isolate AT076 at 20°C and 30°C were found to be greater than isolate AT007. It was observed that isolate AT076 had more spore production with 1.61 × 10^7^ spore/disc at 30°C and 44.33% germination after UV radiation for 15 min. The numbers of spores per 5 mm disk area for isolates AT076 and AT007 were found to be 1.2 × 10^6^ and 1.0 × 10^6^. These results show that isolate AT076 was more virulent and more UV-tolerant and had higher tendency to mass production compared to isolate AT007 against* T. urticae*. As a result of this study, isolate AT076 can be used in the biocontrol as mycopesticide.

## 1. Introduction

*T. urticae* is generally a pest for the agricultural areas and it has also economic damage [1]. The chemical insecticides used in the control of* T. urticae* have been reported [2, 3]; however, these applications are not preferred because of their negative effects on the environment. The entomopathogenic fungi can be seen to be an important alternative to the management of various arthropod species and they are arguably the best for the environment [4]. The use of* Beauveria *spp. has an increasing tendency to control mites [5–7].* B. bassiana* (Bals.) Vuill. is a well-known mycopesticide and is considered to be one of the promising entomopathogenic fungi [8].* Beauveria* genus was shown to be affective against members of the insect orders Coleoptera, Lepidoptera, and Hymenoptera [9, 10]. In addition, the studies have revealed* B. bassiana* to be an excellent pathogen of Acari, especially the two-spotted spider mite,* T. urticae* Koch [10–12]. Success of commercial mycopesticides including entomopathogenic fungi in the environment has been limited when compared with conventional insecticides because they are sensitive to UV radiation [13, 14]. The conidial inactivation caused by UV radiation is expected to reduce the efficiency of mycopesticides [15]. However, the increasing of permanence of these fungi in the environment may be possible through selection of isolates with high UV tolerance [16]. Mycopesticides' tendency to mass production is one of the factors limiting their commercial use [17]. In this case, the formulations containing the selected isolates may be important in the development of mycopesticides and may have a commercial advantage compared to others [18]. In addition, selected isolates for biological control may have a promising potential for use in the different biotechnological applications [19].

The first step in selection of candidate biological control agent is laboratory evaluation of the effectiveness. The most virulent isolates to a pest are isolated from the same or related species [20]. In the present study, we selected two local* B. bassiana* isolates derived from* T. urticae* and evaluated their pathogenicity potentials towards* T. urticae*. Furthermore, mycelial growth and sporulation of isolates in different temperatures, tolerance against UV radiation, and tendency to mass production were compared in order to select the most suitable isolate as mycopesticide.

## 2. Materials and Methods

### 2.1. Biological Materials

The stock culture of* T. urticae* was reared on bean plants at 25 ± 1°C and 60 ± 5% relative humidity (RH) with a 12 : 12 h (L : D) photoperiod. To obtain fixed-age individual, detached leaf system was prepared as indicated by Shi and Feng [5]. Then, mite eggs were allowed to grow and develop for 15 days. AT007 and AT076 isolates of* B. bassiana* were used in this study. These isolates were previously isolated from* T. urticae* [21, 22] and maintained for storage in the slant agar medium containing Sabouraud dextrose agar (SDA) at 4°C.

### 2.2. Conidial Suspensions and Viability

The isolates were cultured in SDA containing 2% yeast extract and kept for two weeks at 25°C for conidial production. Conidia were suspended in the sterile distilled water, a surfactant (0.2 ml/l Tween 80) was added to reduce clumping of the conidia, and finally the suspensions were vortexed to get a homogenous state [23]. The prepared suspensions were then filtrated through three layers of muslin to eliminate hyphae and unsuspended conidia. The spore concentrations were determined by using a haemocytometer and suspensions were prepared in the logarithmic series from 1 × 10^6^ to 1 × 10^8^ [24].

Conidial viability was tested in SDA plates. 0.1 ml was taken from suspensions in the sterile conditions and spread over the SDA medium. After 24 h, percent germination was determined from 100 spore counts on each plate [25]. Only conidia with a germ tube as long as the conidium's width were considered to be germinated. Spore suspensions were sealed with Parafilm and kept at 4°C in a refrigerator until use [26].

### 2.3. Effect of Conidial Concentration and RH

A dose-mortality bioassay of* B. bassiana* AT007 and AT076 isolates was conducted to select the most virulent isolate [27]. For each isolate, 1 ml of three different aqueous suspensions (1 × 10^6^, 1 × 10^7^ and 1 × 10^8^) supplied with 0.02% Tween 80 was sprayed onto downside of bean leaf discs (30 mm diameter) by using a hand sprayer. The leaf discs were dried in the air and placed in Petri dishes. Distilled water containing 0.02% Tween 80 was used as control. Twenty-five fixed-age mites arbitrarily taken from stock culture were transferred to each disc by means of a fine soft brush. After exposure, all Petri dishes were sealed with Parafilm and the holes of 6 mm diameter were opened on the lids for proper ventilation. All Petri dishes were incubated at 25 ± 1°C, 65% and 100% RH. Mortality was recorded on daily basis for a period of 7 days. Dead mites were surface-sterilized in 70% ethanol, dried, and transferred to Petri dishes lined with moist filter paper for 10 days to observe mycosis. Mortality caused by fungi was confirmed by microscopic examination [28, 29].

### 2.4. Determination of Mycelial Growth and Sporulation on the Different Temperature

Three-day-old cultures on SDA media were used for preparing mycelial disc. 5 mm agar disc with mycelia was retrieved with the help of a cork borer and then it was placed in middle of fresh SDA plates and finally incubated at 20°C, 25°C, and 30°C. Radial growth measured on daily basis for a period of 8 days [7] was calculated as described by Cagan and Svercel [30]. To determine sporulation, 5 mm agar discs were randomly taken with the help of a cork borer. These discs were placed in 10 ml of 0.02% Tween 80 solution and vortexed to suspend the spores. Spore concentration was determined by using a Neubauer haemocytometer [31].

### 2.5. Determination of the Tendency to Mass Production

200 g of rice was boiled in 600 ml of distilled water for 45 minutes and then filtered, and the resulting rice porridge was homogenized by mixing. Then, the rice porridge was spread on glass Petri dishes (12 cm diameter) and autoclaved. 5 ml suspension (1 × 10^6^ conidia/ml) as a single dose was sprayed onto rice media by using a hand sprayer and the inoculated plates were incubated at 25°C for 14 days. At the end of this period, 5 mm agar discs were randomly taken with the help of a cork borer. The discs were placed in 10 ml of 0.02% Tween 80 solution and vortexed to suspend the spores. Spore concentration was determined as previously described.

### 2.6. Determination of the Natural Tolerance of* B. bassiana* Isolates to UV Radiation

To compare the UV tolerance, 1 ml of conidial suspensions was spread over the surface of the SDA medium and all plates were exposed to UV irradiance provided by a fluorescent lamp (Philips 35 W), for 0, 15, 30, and 60 minutes from 30 cm distance. Control plates were covered with aluminium foil. After irradiation, the plates were incubated at 25 ± 1°C for 24 h under the dark. Then, one drop of lactophenol blue and a coverslip were placed on the plates and conidial germination was calculated as described by Lee et al. [13].

### 2.7. Identification of the Candidate Isolate

The identification of the* B. bassiana* isolate AT076 was performed by sequencing a fragment of genome. The primers of ITS1 and ITS4 were used for the polymerase chain reaction (PCR) [32]. PCR products were analyzed in 1% agarose gels by horizontal gel electrophoresis. The products were purified by following the protocols of commercial PCR Purification Kit. After purification, ITS rDNA gene was sequenced in both directions at RefGen Co., Ltd., Turkey. The sequences chromatograms were assembled and the sequence was compared to all known sequences in the GenBank by using BLASTN 2.2.26+ program [33] and deposited with the GenBank database under the accession number* MF593119*.

### 2.8. Data Analysis

The experimental design was a randomized complete block with three replicates, and each replicate consisted of 25 mites. The analysis of variance was conducted using one-way ANOVA test using SPSS 15.0. LT_50_ and LT_90_ values were determined with EPA Probit Analysis Program (version 1.5).

## 3. Results and Discussion

Selection of isolates is the first step prior to commercialization of mycopesticides. For this purpose, the effectiveness of candidate biological control agents is evaluated in the laboratory.* B. bassiana* isolates used in this study were previously obtained from Erzurum, East Anatolia. This region, as it is a significantly cold territory at a relatively high altitude, is one of the most important areas of Turkey. Therefore, the present study was undertaken to determine these local isolates potentials in biological control as mycopesticide.

### 3.1. Effect of Conidial Concentration and RH

*B. bassiana* has shown pathogenicity against many insect pests and and it is commercially available as mycoinsectiside [4]. To evaluate new candidate isolates, the optimal conditions such as conidial concentration and RH need to be determined [34]. In viability tests, 95–100% of the spores were germinated and it was found that both tested fungal isolates were pathogenic to the* T. urticae* in a mortality rate between 90.7 ± 5.6% and 100% after 7 days from application. Microscopic investigations confirm that all mites died due to mycosis, and* B. bassiana* was reisolated from all dead mites. The results of mortality of both* B. bassiana* isolates against* T. urticae* with LT_50_ and LT_90_ values are depicted in Table 1.

In bioassays at 65% RH, the differences between mortality rates in the three conidial concentrations were found to be significant (*p* < 0.05) except for the 7th day. An increase in the mortality was found with time and spore concentration. The LT_50_ and LT_90_ values for both isolates vary between 2.50–4.80 days and 5.20–7.82 days, respectively. On the other hand, percentage of pathogenicity for the* T. urticae* in bioassays at 100% RH was the highest among them. Mortality caused by isolate AT076 was found to be significantly different (*p* < 0.05) on the 5th day. For this isolate, the lowest conidial concentration gave the least control (89.3 ± 8.4%) of the mites. The mortality was found to be 93 ± 6% at the intermediate concentration and the best mortality rates as 100% were obtained for the highest conidial concentrations (1 × 10^8^ conidia/ml). On the 7th day, this isolate showed similar virulence to isolate AT007 (*p* < 0.05) for all conidial preparations. Isolate AT076 leads to the shortest LT_50_ value with 2.17 days. This value was significantly (*p* < 0.05) shorter than one of LT_50_ values obtained with AT007. Therefore,* B. bassiana* isolate AT076 was consistently more virulent than AT007 against* T. urticae*. Similarly, the previous studies demonstrated that* B. bassiana* was pathogenic against* T. urticae*, but the levels of mortality were different among the isolates [35, 36].* B. bassiana* isolate AT076 was found to be highly pathogenic against* T. urticae* even when it was exposed to low RH with low conidial concentrations. As known, the lower concentration enables the most cost efficient method to be used in biological control [37]. Fungi require very high humidity to sporulate and germinate. Therefore, low humidity causes a significant reduction in the infectivity of entomopathogenic fungi [4]. In our study, mortality generally decreased under low humidity conditions. The 35% increase in the humidity level caused 8–12% increases in the mortality rate for isolate AT076 on the 3rd and 5th days, respectively. Similarly, the previous studies showed that a decrement in the humidity level caused a decrease in the mortality in the experiments for the different levels [34, 38]. For example, Albayrak Iskender et al. [37] reported that the 35% decrease in the humidity level caused 50.9% decrease in the mortality rate for* Pristiphora abietina* larvae.

### 3.2. Determination of Mycelial Growth and Sporulation on the Different Temperature

Both isolates showed mycelial growth at all the test temperatures and the highest mycelial growth of both isolates was obtained at 25°C. In this temperature, isolates showed similar mycelial growths (*p* < 0.05) and the mycelial growth decreased at the higher or the lower temperatures. Similarly, in a study conducted by Nussenbaum et al. [39], it was shown that mycelial growth of* B. bassiana* and* Metarhizium anisopliae* decreased with changes in the temperature. Additionally, another study showed that mycelial growth of* Beauveria* isolates decreased with an increase in the temperature [40].

Both isolates showed spore production at all the test temperatures and the yield increased with temperature. The highest spore production for isolate AT007 was registered at 25°C and 30°C; however, for isolate AT076, the highest spore production was found to be only at 30°C. In temperature of 30°C, isolates showed similar spore yields (*p* < 0.05), but response of isolates towards temperature was significant in the lower temperatures (*p* < 0.05). This is almost consistent with Uzma and Gurvinder [31].

### 3.3. Determination of the Tendency to Mass Production

Rice was the best substrate for mass production of* B. bassiana* conidia and for this reason it is widely used in the industrial production as solid substrate [41]. Therefore,* B. bassiana* isolates were tested for their maximum spore production on the rice. Among both isolates, more conidia (1.2 × 10^6^ spore/5 mm agar disc) were obtained by isolate ATA076.

### 3.4. Determination of the Natural Tolerance of* B. bassiana* Isolates to UV Radiation

The natural UV tolerance was highly variable for both isolates and* B. bassiana* AT007 is more sensitive to UV radiation for all exposure time. As could be seen from Table 2, isolate AT076 exposed to UV radiation for 15 minutes showed 44.33% germination and isolate AT007 showed 16.58% germination after 24 hours. However, control conidia were 100% germinated after 24 h. The viability of conidia of both isolates decreased with an increase in the exposure time. Similarly, the studies showed that* B. bassiana* had varying tolerance towards UV and their response was affected by exposure time [16, 42].

## 4. Conclusion

The usage of mycopesticides as part of an integrated pest management (IPM) strategy could reduce the dependence on the chemical control. In the present study, we selected two local* B. bassiana* isolates and evaluated their pathogenicity potentials towards* T. urticae.* As a conclusion of this study,* B. bassiana* isolate AT076 was consistently more virulent than AT007 against* T. urticae*. Furthermore, this isolate was found to be more UV-tolerant and had higher tendency to mass production. The present results suggest that isolate AT076 has good potential as a mycopesticide within an IPM program.

## Figures and Tables

**Table 1:** Mortality of *B. bassiana* isolates against *T. urticae* with LT_50_ and LT_90_ values^*∗*^.

		Dose	3rd day	5th day	7th day	LT_50_	Confidence limits (%95)	LT_90_	Confidence limits (%95)
65 ± 5% RH	AT007	1 × 10^6^	24 ± 2.3^b^	62.7 ± 6.4^b^	90.7 ± 5.6^a^	4.80^a^	3.62–4.57	7.71^a^	6.53–10.14
		1 × 10^7^	30.7 ± 4.3^ab^	68 ± 4.0^ab^	92 ± 6.3^a^	3.73^b^	3.25–4.24	7.82^a^	6.49–10.61
		1 × 10^8^	37.3 ± 5.3^a^	76 ± 8.2^a^	96 ± 0.0^a^	3.34^c^	2.90–3.77	6.58^b^	5.61–8.42
	AT076	1 × 10^6^	49.3 ± 4.3^b^	76 ± 6.4^b^	100 ± 0.0^a^	2.86^a^	2.42–3.29	6.36^a^	5.32–8.36
		1 × 10^7^	53.3 ± 4.7^b^	85.3 ± 5.0^a^	100 ± 0.0^a^	2.71^a^	2.30–3.11	5.75^b^	4.88–7.34
		1 × 10^8^	60 ± 7.3^a^	88 ± 8.2^a^	100 ± 0.0^a^	2.50^b^	2.11–2.87	5.20^b^	4.44–6.54
		Control	1.33 ± 1.3^c^	1.33 ± 1.3^c^	4 ± 2.3^b^				

100% RH	AT007	1 × 10^6^	34.7 ± 3.3^b^	73.3 ± 7.4^b^	100 ± 0.0^a^	3.33^a^	2.90–3.74	6.45^a^	5.53–8.17
		1 × 10^7^	36 ± 5.3^ab^	78.7 ± 6.0^ab^	100 ± 0.0^a^	3.16^ab^	2.24–4.00	6.08^b^	4.68–7.61
		1 × 10^8^	44 ± 4.3^a^	89.3 ± 7.2^a^	100 ± 0.0^a^	2.89^b^	2.50–3.24	5.19^c^	4.54–6.30
	AT076	1 × 10^6^	48 ± 5.3^b^	89.3 ± 8.4^c^	100 ± 0.0^a^	2.77^a^	2.37–3.16	5.61^a^	4.80–7.05
		1 × 10^7^	65.3 ± 5.7^a^	93.3 ± 6.0^b^	100 ± 0.0^a^	2.26^b^	1.90–2.60	4.53^b^	3.89–5.61
		1 × 10^8^	68 ± 6.3^a^	100 ± 0.0^a^	100 ± 0.0^a^	2.17^b^	1.83–2.47	4.04^c^	3.50–4.93
		Control	1.33 ± 1.3^c^	2.67 ± 1.3^d^	5 ± 2.3^b^				

^*∗*^All values represent mean ± standard error of three determinations (*n* = 3). Same alphabet letters in the same column are not significantly different at *p* < 0.05.

**Table 2:** Mycelial growth and sporulation of isolates in different temperatures, tolerance against UV radiation, and tendency to mass production^*∗∗*^.

Isolates	Mycelial growth (mm)			Spore production (×10^7^)			UV tolerance (viability %)				Mass production (×10^6^)
	20°C	25°C	30°C	20°C	25°C	30°C	Control	15 min.	30 min.	60 min.	
AT007	1.81^bB^	2.87^aA^	1.49^cB^	1.20^bA^	1.50^aA^	1.52^aA^	100^aA^	16.58^bB^	3.33^cB^	2.16^cB^	1.0^b^
AT076	2.35^cA^	2.87^aA^	2.61^bA^	1.03^cB^	1.32^bB^	1.61^aA^	100^aA^	44.33^bA^	13.4^cA^	12.0^cA^	1.2^a^

^*∗∗*^All values represent mean ± standard error of three determinations (*n* = 3). Different lowercase letters in the same row and different uppercase letters in the same column indicate significant differences (*p* < 0.05).
